# A Comparative Analysis of Molecular Epidemiology and Antibiotic Resistance in Group B Streptococcus Isolated From Invasive and Noninvasive Adult Cases in Daejeon, South Korea

**DOI:** 10.1155/cjid/8797954

**Published:** 2025-08-29

**Authors:** Myoung-Schook Yoou, Ji Hun Jeong, Chunhwa Ihm

**Affiliations:** Department of Laboratory Medicine, Daejeon Eulji Medical Center, Eulji University, Daejeon, Republic of Korea

**Keywords:** antimicrobial susceptibility tests, capsular polysaccharide, Group B streptococcus, multilocus sequence typing, pilus islands

## Abstract

**Background:** This study aims to determine the molecular features and antimicrobial resistance of *Streptococcus agalactiae* (Group B streptococcus, GBS) causing invasive and noninvasive infections in Korean adults.

**Methods:** Sequence type (ST), capsular serotype, pilus island typing, and antimicrobial susceptibility were analyzed for GBS isolates obtained at a hospital laboratory that processed the primary clinical specimens collected from Korean adults between 2021 and 2024.

**Results:** Among the 90 isolates, Serotype VIII (34.4%) was the most common, followed by V (17.8%), Ia (12.2%), VI (11.1%), II (10.0%), and Ib (6.7%). Every isolate contained at least one PI gene, of which PI2a (37.8%) was the most frequently observed combination. The combination of PI1 and PI2b was present in 34.4% of isolates, followed by PI1 and PI2a (24.4%) and PI2b alone (3.3%). Among the 18 STs identified, the capsular serotype VIII/ST2–clonal complex 1 (CC1) was dominant (34.4%), followed by V or VI/ST1 (12.2%). All isolates were susceptible to both ampicillin and vancomycin, while antibiotic resistances were observed for erythromycin (27.8%), levofloxacin (13.3%), clindamycin (25.6%), and tetracycline (47.8%), respectively.

**Conclusions:** These data are key elements to help design prevention and treatment strategies for GBS infection in Korean adults.

## 1. Introduction

Group B Streptococcus (GBS), typically found as a harmless inhabitant of the gastrointestinal and genitourinary tracts, can, nonetheless, provoke invasive infections in neonates, expectant mothers, and individuals with preexisting medical conditions [1]. GBS colonization in the vagina during pregnancy holds clinical significance as it correlates with neonatal meningitis and septicemia [2]. Although GBS disease has traditionally been considered a condition primarily affecting neonates and pregnant women, its incidence is increasing among nonpregnant adults [3]. In recent decades, there has been a surge in GBS infections among adults, notably those grappling with underlying ailments such as diabetes [2]. The evidence indicates a striking uptick in GBS infections among adults and the elderly, particularly those battling diabetes mellitus, cancer, undergoing renal dialysis, or having other significant underlying health issues. The estimated mortality rate due to severe GBS infections in the elderly hovers around 15% [4].

The primary virulence factor of GBS is its capsular polysaccharide (CPS), coded by CPS gene. Each serotype possesses different disease-causing capabilities [5]. CPS aids in the release of virulence factors in human GBS isolates, enabling the microorganisms to evade host defense mechanisms [6]. Given the high morbidity and mortality associated with invasive GBS infections, there is a concerted research effort toward developing a multivalent conjugate polysaccharide vaccine. Hence, understanding the distribution and alterations in GBS serotypes across various populations is imperative [7]. GBS pili may heighten colonization and infection by facilitating bacterial adhesion to host cells, penetrating endothelial and epithelial barriers, and resisting bacterial ingestion and elimination by host phagocytes [8]. The widespread presence of pilus islands in GBS isolates renders these structures promising vaccine candidates against infections [9]. GBS remains largely susceptible to beta-lactam antibiotics, such as penicillin; however, increasing resistance rates to second-line agents, including erythromycin and clindamycin, has been reported. Moreover, there is a growing trend of resistance to other antibiotic classes, such as fluoroquinolones and aminoglycosides, underscoring the need for continued antimicrobial surveillance and resistance monitoring [10].

Clinical manifestations and serotype distribution in adults contrast with those observed in neonatal infections [11]. Continuous surveillance of the molecular features and antimicrobial resistance of infectious GBS is crucial for both the prevention and management of GBS-related infections. This study aimed to scrutinize the molecular features and antimicrobial resistance profiles of GBS isolated from adult patients with either invasive or noninvasive bacterial infections over approximately 3 years at Daejeon in Korea.

## 2. Materials and Methods

### 2.1. GBS Isolates

All adult patients from whom GBS strains were identified at a single hospital between February 2021 and May 2024 were retrospectively reviewed for limited, anonymized data including age, sex, specimen type, and isolate source. No access to individual medical records was required, and the analysis was conducted solely on bacterial isolates obtained from routine clinical specimens. As this study involved no direct interaction with patients and did not include identifiable personal information, ethical approval was not required. The study cohort consisted of adults aged 20 years and older, of whom 56.7% (51 out of 90) were female, and the remainder was male. Invasive GBS isolates were defined as those obtained from normally sterile sites such as blood, tissue, or closed pus. GBS isolates obtained from sites such as vaginal discharge, urine, open pus, or ear discharge were considered noninvasive.

### 2.2. Multilocus Sequence Typing (MLST)

MLST analysis was conducted by sequencing seven housekeeping genes (*adhP, atr, glck, glnA, pheS, sdhA,* and *tkt*) following established protocols [12]. Sequence types (STs) were assigned by querying the *S. agalactiae* MLST database (http://pubmlst.org/sagalactiae). Any alleles and STs that were novel were submitted to the *S. agalactiae* MLST database. Clonal complex (CC) analysis was performed using the entire GBS MLST database and goeBURST [13]. STs and CCs were determined based on the database information, and the correlation between CCs and serotypes was utilized to construct a minimum spanning tree using Phyloviz software [14]. The goeBURST distance score is a method used to measure the genetic distance between different STs, particularly in the context of MLST data. It quantifies the genetic differences between bacterial strains by assessing the variation in their allelic profiles across multiple loci.

### 2.3. Capsular Serotyping

The CPS types were identified utilizing PCR amplification and sequencing of the CPS type—specific regions within the CPS locus for Serotypes Ia, Ib, and II through VIII [15]. Low-frequency Serotype IX is not included in the protocol, and those isolates that could not be typed were categorized as nontypeable (NT).

### 2.4. Pilus Islands Typing

We designed PCR assays targeting the sortase genes to detect the presence of pilus islands in each isolate by directly assessing the size of the amplification product. The oligonucleotide primers, their respective target sites, and sequences are detailed as previously outlined [16]. PCR reactions were conducted for each bacterial isolate using the following primers: PI1-UP, PI1-DN, PI2a-UP and PI2a-DN, and PI2b-UP and PI2b-DN. All PCR reactions were performed using Bio-Rad CFX96 instruments.

### 2.5. Antimicrobial Susceptibility Tests

Antibiotic susceptibility testing was performed using the VITEK 2 AST ST-03 card (bioMerieux), and the results were interpreted based on the current Clinical and Laboratory Standards Institute (CLSI) guidelines [17]. A panel of antibiotics, including ampicillin, chloramphenicol, clindamycin, ceftriaxone, erythromycin, levofloxacin, linezolid, moxifloxacin, benzylpenicillin, trimethoprim/sulfamethoxazole, cefotaxime, tetracycline, tigecycline, and vancomycin, was tested. Only isolates categorized as resistant were included in resistance rate calculations; intermediate isolates were excluded.

### 2.6. Statistical Analysis

Fisher's exact test was performed to evaluate the association between antibiotic resistance and invasive versus noninvasive group types for each antibiotic. Resistance was classified as either resistant (1) or nonresistant (0), and 2 × 2 contingency tables were constructed for each antibiotic to assess the significance of the differences.

## 3. Results

### 3.1. Laboratory Findings

During the course of this study, 90 cases of invasive and noninvasive GBS infections involved individuals over 19 years of age.  Table 1 illustrates the distribution of GBS strains and the average age of patients based on the type of test subjects included in this study. The median age of the 90 subjects was 59.4 years, with 51.1% (46 out of 90) being over 60 years. Among the 90 patient samples analyzed, the most common source of isolates was random urine (32.2%), followed by blood (21.1%), vaginal discharge (14.4%), open pus (10.0%), closed pus (10.0%), tissue (4.4%), other sources (4.4%), sputum (2.2%), and ear discharge (1.1%). Noninvasive GBS infection was confirmed in 64.4% GBS isolates, while 35.6% were attributed to invasive infection (Table 1).

### 3.2. MLST

**Figure 1:**
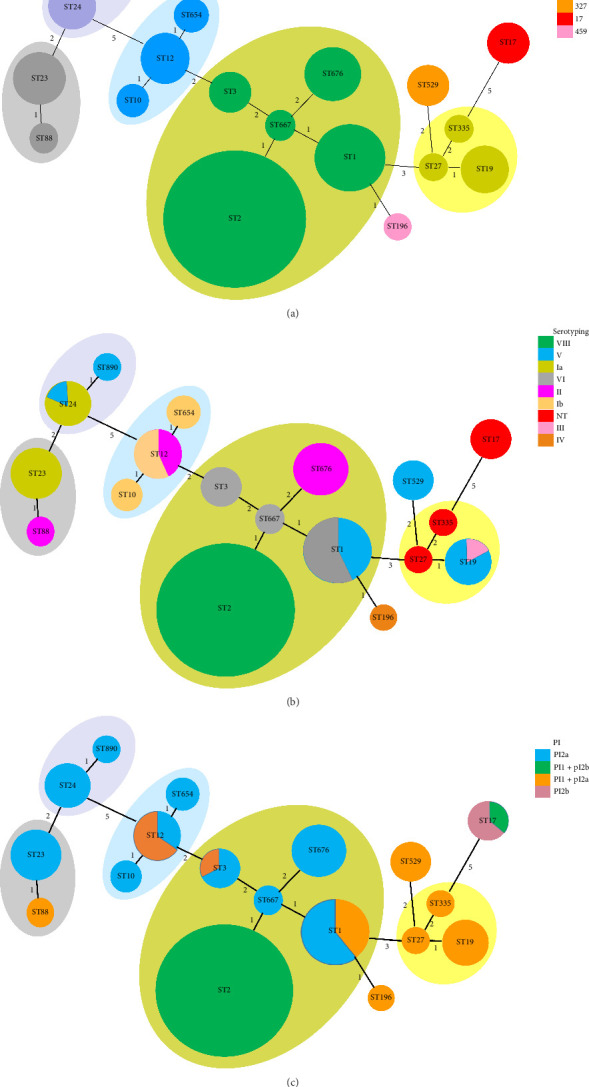
The minimum spanning tree (MST) of the 90 GBS isolates depicted the relationships among clonal complexes (CC), sequence types (ST), serotypes, and pilus island types (PI). In the diagram, STs are shown as circles, with their sizes proportional to the number of isolates. CC (a), ST (b), and PI (c) were differentiated by distinct colors. STs within the same clonal complex were shaded uniformly. Numerical values from 1 to 5 represented the goeBURST distance score, a number that represents the genetic difference between two sequence types (STs) by quantifying how many MLST genes differ between them.

In total, we identified 18 different STs among the 90 invasive and noninvasive GBS isolates. The most prevalent CCs were CC1 (comprising STs: 1, 2, 3, 667, and 676; 57.8%), followed by CC12 (comprising STs: 10, 12, and 654; 8.9%), CC19 (comprising STs: 19, 27, and 335; 8.9%), CC23 (comprising STs: 23,88; 8.9%), CC452 (comprising STs: 24,890; 6.7%), CC327 (comprising STs: 529; 4.4%), CC17 (comprising STs: 17; 3.3), and CC459 (comprising STs: 196; 1.1%). The dominant capsular serotype expressed by ST2 (34.4%) was Serotype VIII, which was found at high rates in both invasive and noninvasive isolates. ST1 (12.2%) expressed Serotypes V and VI. Further details on CC and serotype distribution are provided in Table 2 and Figure 1.

### 3.3. GBS Serotype

In our study, we found eight serotypes among the 90 GBS isolates. The most prevalent serotype was Serotype VIII, accounting for 34.4% (31 out of 90) of all isolates. Following that, Serotype V accounted for 17.8% (16 out of 90) of all isolates, Serotypes Ia and VI each represented 12.2% and 11.1% (11 out of 90 and 10 out of 90) of all isolates, while Serotype II accounted for 10.0% (9 out of 90) of all isolates. Serotype Ib constituted 6.7% (6 out of 90) of all isolates, whereas Serotypes III and IV were rarely discovered, each representing 1.1% of all isolates. Additionally, 5.6% (5 out of 90) of all isolates were NT (Table 2).

### 3.4. PI Type

All strains were found to carry either PI2a or PI2b, with the PI1 locus detected in 58.8% of the strains. The most frequently observed pilus island was PI2a alone (36.7%), followed by combinations of PI1 + PI2b (35.6%), PI1 + PI2a (25.6%), and PI2b alone (2.2%). Specifically, all Serotype VIII isolates (31 out of 31, 100.0%) contained only the PI1 + PI2b island, and the PI1 + PI2b island was the most prevalent in both invasive and noninvasive isolates. Further details on PI and ST distribution can be found in Table 3.

### 3.5. Antibiotic Sensitivity Profile

All isolates exhibited sensitivity to beta-lactam antibiotics (ampicillin, ceftriaxone, and cefotaxime) and vancomycin. However, resistance to clindamycin, erythromycin, levofloxacin, and moxifloxacin was observed in 25.6%, 27.8%, 13.3%, and 11.1% of the isolates, respectively. Notably, nearly all erythromycin-resistant strains (with the exception of four) also demonstrated resistance to clindamycin. Particularly noteworthy was the widespread resistance (47.8%) to tetracycline among GBS isolates. Detailed resistance profiles are provided in Table 4. According to Fisher's exact test, resistance to levofloxacin and moxifloxacin differed significantly between invasive and noninvasive isolates (*p* < 0.05), whereas no significant differences were found for clindamycin, erythromycin, or tetracycline (*p* > 0.05), indicating comparable resistance levels between the two groups for these antibiotics.

## 4. Discussion

While GBS typically resides as part of the commensal flora in the genital and lower gastrointestinal tract of healthy adults, its vaginal colonization during pregnancy can have significant clinical implications. Over recent decades, GBS infections have seen a surge in prevalence among adults, particularly those with underlying medical conditions such as diabetes. In our present study, we aimed to analyze the molecular characteristics, the presence of pilus island as an important virulence factor, and serotype distribution of invasive and noninvasive GBS in Korea. Additionally, all isolates underwent antibiotic resistance testing to determine the frequency of resistance to selected antibiotics.

Serotype determination has traditionally been a cornerstone of epidemiological studies on GBS and is pivotal in the development of broadly protective vaccines containing capsular polysaccharides or polysaccharides conjugated to proteins [18]. Available data suggest that the serotype distribution of GBS isolates remains largely consistent across different regions worldwide over the past 30 years, with five serotypes (Ia, Ib, II, III, and V) predominantly associated with disease. Thus, conjugate vaccines including some or all of these serotypes hold significant promise for preventing this disease [6]. Studies conducted in Brazil have typically found Serotypes Ia, II, III, or V to be most prevalent [19]. A study by Lachenauer et al. highlighted a high prevalence of Serotypes VI and VIII among colonizing GBS strains isolated from pregnant women in Japan. They reported that a high prevalence of Serotypes VI and VIII among colonizing GBS strains was isolated from pregnant women in Japan between May 1992 and June 1994 [20, 21]. In Korea, predominant GBS serotypes shifted from Ib (48.3%), Ia (24.1%), and III (20.7%) in 1995 to III (29.6%), V (22.2%), and VI (22.2%) in 2008-2009 and further to V (22.7%), VIII (20.0%), and III (20.0%) in 2017–2019 [22]. While Serotype VIII is known to effectively colonize and invade human endothelial cells, it exhibits lower capability in inducing the release of inflammatory cytokines such as TNF-α and IL-10 compared to Serotype III [23, 24]. In our study, we found that Serotype VIII (34.4%) was the most common, followed by V (17.8%), Ia (12.2%), VI (11.1%), II (10.0%), and Ib (6.7%). Notably, the prevalence of Serotype VIII observed in our study was higher than those reported in other studies. According to Choi et al., 34.2% of GBS isolates in Korea were associated with invasive infections, a figure similar to our finding of 35.6% [25]. However, the most common capsular serotypes among all isolates in their study differed from ours, with Serotype III (39/120, 32.5%), followed by V (20/120, 16.7%) and VIII (20/120, 16.7%). These findings highlight the similarities in the prevalence of invasive GBS infections in Korea while also indicating notable differences in the distribution of capsular serotypes between our study and that of Choi et al. While most GBS isolates can be classified into Serotypes Ia, Ib, and II through VIII, a small percentage (4%–7%) is NT [26]. Similarly, in our results, 5.6% could not be classified.

MLST analyses of GBS isolates from various countries have revealed that only a limited number of CCs, including CC1, CC10, CC17, CC19, and CC23, are associated with colonizing or invasive isolates [27]. Notably, CC23, CC17, and CC1 accounted for 67.2% of isolates detected in Europe and the United States [28]. Among these, CC17 is recognized as a major hypervirulent MLST clone currently circulating globally [29]. As of April 2022, PubMLST contained 43 isolates with a capsular serotype and/or genotype of VIII, the majority of which were part of ST1 (CC1) (34/43 isolates) [30]. ST2 Serotype VIII isolates were limited within the database, with only four isolates identified in Australia, of which three appeared to be invasive being isolated from blood and joint fluid [31]. In Korea, the Serotype III/ST17 strain was the most common in invasive GBS isolates, while the Serotype VIII/ST2 strain was the most prevalent in noninvasive GBS [25]. In our study, Serotype VIII (31/90, 34.4%), represented by ST2 and belonging to CC1, was the most prevalent among both invasive (13/31, 41.9%) and noninvasive (18/31, 58.1%) GBS isolates in Daejeon, Korea. The hypervirulent MLST clone ST17 was identified infrequently, with a prevalence of 3.3%. ST17 is known to be closely associated with late-onset disease (LOD) and meningitis in neonates [32], and its low prevalence in this study is likely attributable to the adult population investigated. This finding stands in marked contrast to neonatal GBS infections, where ST17 is commonly detected and highlights the distinct differences in GBS strain distribution between adult and neonatal populations.

Pili are essential structures for GBS adhesion, primarily involved in epithelial cell colonization, biofilm formation, translocation, and invasion. Unlike Gram-negative bacteria, pili in GBS are cell wall–anchored appendages extending from the bacterial surface [33, 34]. Rinaudo et al. discovered that the majority of biofilm-forming GBS strains carry PI-2a. Through the use of insertion and deletion mutants, it was confirmed that PI- 2a, rather than PI-1 and PI-2b, is responsible for conferring the biofilm-forming phenotype [35]. Furthermore, PI-2b has been proposed to play a unique role in promoting strain invasiveness and interactions with bacterial host cells. Mutants lacking PI-2b exhibit reduced adherence and invasion capacities for epithelial and endothelial cells [36]. In a study conducted by Rinaudo et al. in Italy, it was found that 59.3% of GBS isolates harbor PI1 + PI2a genes [35]. Similarly, Martins et al. in Spain reported that the majority of isolates contained PI1 + PI2a, with PI2b being the least prevalent (0.6%). Various studies have consistently shown that the majority of detected pili genes in GBS strains are associated with PI2a [37]. In our study, the most frequently encountered pilus islet was PI-2a alone (33/90, 36.7%), followed by combinations of PI1 + PI2b and PI1 + PI2a, while PI2b was observed at the lowest frequency.

Penicillin and ampicillin are considered the primary treatment options for human *S. agalactiae* infections. For individuals allergic to penicillin, erythromycin and clindamycin are recommended alternatives. In general, GBS is widely regarded to be 100% susceptible to penicillin and ampicillin [38]. Additionally, our research findings confirmed that GBS isolates were uniformly sensitive to beta-lactam antibiotics, including ampicillin, ceftriaxone, and cefotaxime. However, contrasting with the low resistance rates in European countries (1.4%–1.5%), a notable prevalence of resistance to levofloxacin (35.7%–42.0%) has been documented in Asia–Pacific countries [39]. In this study, the average resistance rates of our GBS isolates to clindamycin, erythromycin, levofloxacin, and tetracycline were 25.6%, 27.8%, 13.3%, and 47.8%, respectively. Based on a comprehensive analysis of 334 studies conducted across 57 countries, significant regional differences and temporal trends in antibiotic resistance were identified [40]. This study exclusively targeted nonpregnant adult populations: Therefore, the findings may not be directly applicable to neonatal or maternal GBS cases. Nonetheless, the data offer valuable insights into the molecular epidemiology and antimicrobial resistance profiles of GBS in Korean adults, a group that is currently underrepresented in the literature. To effectively manage GBS infections, it is crucial to maintain ongoing surveillance and investigate region- and time-specific patterns of antibiotic resistance. Such efforts will enable the development of tailored treatment strategies and support the implementation of systematic measures to curb the spread of resistant strains.

## 5. Conclusion

In conclusion, this study provides important insights into the molecular characteristics and antimicrobial resistance profiles of invasive and noninvasive GBS isolates collected from adults in Korea. During the study, eight serotypes were detected, with Serotype VIII comprising 34.4% of isolates, predominantly carried by CC1 (ST2) and PI-2b. While all isolates were sensitive to beta-lactam antibiotics, resistance to clindamycin and erythromycin was observed in 25.6% and 27.8% of isolates, respectively, with tetracycline resistance notably at 47.8%. Understanding the epidemiological features of GBS is crucial for effective prevention strategies and the development of appropriate treatment protocols in the future. However, it is important to acknowledge the limitations of our study, particularly its reliance on data from a single center with limited geographical coverage. Therefore, to provide a more comprehensive understanding of GBS epidemiology in Korea, periodic studies involving multiple centers, including primary clinics, are needed.

## Figures and Tables

**Table 1:** Patient demographics and sample-type characteristics in 90 GBS isolates.

Aspect	Number (percentage %)
Sex	
Male	39 (43.3%)
Female	51 (56.7%)
Age	
< 60 years	44 (48.9%)
≥ 60 years	46 (51.1%)
Sample type	
Invasive infection	32 (35.6%)
Blood	19 (21.1%)
Closed pus	9 (10.0%)
Tissue	4 (4.4%)
Noninvasive infection	58 (64.4%)
Random urine	29 (32.2%)
Vaginal discharge	13 (14.4%)
Open pus	9 (10.0%)
Others	4 (4.4%)
Sputum	2 (2.2%)
Ear discharge	1 (1.1%)

**Table 2:** Relationship between serotypes, clonal complexes, and sequence types of the 90 Group B Streptococcus isolates.

CC	ST	*n* (%)	Serotype (*n*)	
			Invasive isolates	Noninvasive isolates
CC1		52 (57.8%)		
	ST1	11 (12.2%)	VI (3)	V (5), VI (3)
	ST2	31 (34.4%)	VIII (13)	VIII (18)
	ST3	3 (3.3%)	VI (1)	VI (2)
	ST667	1 (1.1%)		VI (1)
	ST676	6 (6.7%)		II (6)
CC12		8 (8.9%)		
	ST10	1 (1.1%)		Ib (1)
	ST12	5 (5.6%)	Ib (2), II (2)	Ib (1)
	ST654	2 (2.2%)	Ib (1)	Ib (1)
CC17		3 (3.3%)		
	ST17	3 (3.3%)		NT (3)
CC19		8 (8.9%)		
	ST19	6 (6.7%)	V (1)	III (1), V (4)
	ST27	1 (1.1%)		NT (1)
	ST335	1 (1.1%)		NT (1)
CC23		8 (8.9%)		
	ST23	7 (7.8%)	Ia (3)	Ia (4)
	ST88	1 (1.1%)	II (1)	
CC327		4 (4.4%)		
	ST529	4 (4.4%)	IV (2)	IV (2)
CC452		6 (6.7%)		
	ST24	5 (5.6%)	Ia (2)	Ia (2), V (1)
	ST890	1 (1.1%)	V (1)	
CC459		1 (1.1%)		
	ST196	1 (1.1%)		IV (1)

**Table 3:** Distribution between serotype and pilus island of the 90 GBS isolates.

Serotype	PI2a (i)	PI2b (i)	PI1 + PI2a (i)	PI1 + PI2b (i)	Total (i)
Ia	11 (5)				11 (5)
Ib	5 (3)		1 (0)		6 (3)
II	6 (0)		3 (3)		9 (3)
III			1 (0)		1 (0)
IV			1 (0)		1 (0)
V	2 (1)		14 (3)		16 (4)
VI	9 (3)		1 (1)		10 (4)
VII					0
VIII				31 (13)	31 (13)
NT		2 (0)	2 (0)	1 (0)	5 (0)
Total	33 (12)	2 (0)	23 (7)	32 (13)	90 (32)

*Note:* i: invasive GBS number.

**Table 4:** Antibiotic resistance testing of the 90 GBS isolates.

Antibiotics	Invasive isolates (%)	Noninvasive isolates (%)	Total (%)
Clindamycin	7 (7.8%)	16 (17.8%)	23 (25.6%)
Erythromycin	8 (8.9%)	17 (18.9%)	25 (27.8%)
Levofloxacin	2 (2.2%)	10 (11.1%)	12 (13.3%)
Moxifloxacin	1 (1.1%)	9 (10.0%)	10 (11.1%)
Tetracycline	14 (15.6%)	29 (32.2%)	43 (47.8%)

*Note:* All isolates were susceptible to beta-lactam antibiotics (ampicillin, ceftriaxone, and cefotaxime) and vancomycin.

## Data Availability

The data used to support the findings of this study are available from the corresponding author upon reasonable request.
